# Perceptions of Monica Geller in *Friends*: A Pilot Study on Personality Frameworks and Parasocial Relationships

**DOI:** 10.3390/bs15020146

**Published:** 2025-01-29

**Authors:** Danilo Garcia

**Affiliations:** 1 Department of Social Sciences, University of Stavanger, 4021 Stavanger, Norway; danilo.garcia@icloud.com; 2 Promotion of Health and Innovation for Well-Being (PHI-WELL), Department of Social Sciences, University of Stavanger, 4021 Stavanger, Norway; 3 Department of Behavioral Sciences and Learning, Linköping University, 581 83 Linköping, Sweden; 4 Lab for Biopsychosocial Personality Research (BPS-PR), International Network for Well-Being; 5 Centre for Ethics, Law and Mental Health (CELAM), University of Gothenburg, 405 30 Gothenburg, Sweden; 6 Department of Psychology, University of Gothenburg, 405 30 Gothenburg, Sweden

**Keywords:** parasocial relationships, Big Five, HEXACO, Biopsychosocial Model, Monica Geller, media psychology, Multidimensional Measure of Parasocial Relationships

## Abstract

This pilot study investigated how viewers perceive Monica Geller’s personality using three evidence-based personality models: Big Five, HEXACO, and Cloninger’s Biopsychosocial Model. Additionally, it examined how these perceptions are associated to audiences’ engagement in parasocial relationships with this iconic character from the sitcom *Friends*. A sample of sixty-three participants assessed Monica’s personality by responding to the Big Five Inventory (BFI), the HEXACO-60, and the Temperament and Character Inventory (TCI-60). Participants also completed the Multidimensional Measure of Parasocial Relationships (MMPR). Personality scores were contextualized against U.S. population norms (*N*_BFI_ = 711, *N*_HEXACO_ = 1126, *N*_TCI_ = 1948) and Pearson correlations were conducted to explore associations between personality traits and the Affective, Behavioral, Cognitive, and Decisional dimensions of parasocial engagement. Normative comparisons revealed Monica’s perceived Openness and Agreeableness in the Big Five and her Openness and Agreeableness in the HEXACO as significantly below average, while her Big Five Neuroticism and her HEXACO Conscientiousness were significantly above average. In the Biopsychosocial Model, Monica’s Persistence was significantly higher than population norms, while Cooperativeness was significantly lower. Big Five Agreeableness showed correlations across all parasocial engagement dimensions. HEXACO Emotionality was strongly linked to the Affective and Behavioral dimensions, while Honesty–Humility was associated with Cognitive parasocial engagement. In the Biopsychosocial Model, Reward Dependence and Cooperativeness were associated with Cognitive and Affective parasocial engagement, while Self-Directedness was linked to the Behavioral dimension. The Biopsychosocial Model offered the most comprehensive insights, capturing the multidimensional nature of viewer–character engagement. The Big Five and HEXACO models added valuable perspectives, particularly in explaining that traits associated with trust and kindness are linked to decision making. These findings emphasize the importance of integrating multiple personality frameworks to advance the understanding of parasocial relationship engagement, shedding light on the nuanced ways personality traits shape audience perceptions and relationships with media characters, with significant implications for media psychology and personality research. Limitations and avenues for future developments are discussed, building on the insights from this pilot study.

## 1. Introduction

*“Welcome to the real world. It sucks. You’re gonna love it.”*

Monica Geller. (1994–2004). *Friends* [Television series]. In D. Crane, M. Kauffman, K. Bright (Executive Producers). NBC.

In psychological research, structured models of personality have provided essential frameworks for understanding individual differences and their influence on behavior, relationships, and perceptions (Garcia, 2024). The Big Five (John et al., 1988), HEXACO (Ashton et al., 2004a, 2004b) and Cloninger’s Biopsychosocial Model (C. R. Cloninger et al., 1993) represent three distinct approaches to studying personality. The Big Five and HEXACO models, rooted in the lexical paradigm, emphasize broad descriptive traits such as Conscientiousness and Agreeableness, with the HEXACO model adding Honesty–Humility (Ashton et al., 2006; Ashton & Lee, 2007; K. Lee & Ashton, 2004). In contrast, the Biopsychosocial Model delves deeper into the biological, psychological, and social processes shaping both temperament and character traits, emphasizing their dynamic organization within the individual (Cervone, 2004, 2005; C. R. Cloninger & Zwir, 2022; del Val et al., 2024; Zwir et al., 2021). These models not only enhance our understanding of real-life personalities but also offer valuable insights into the perception of fictional media characters, who often serve as relatable figures in shaping audience attitudes and behaviors (So et al., 2024). By integrating these frameworks, this pilot study seeks to explore the personality profile of a specific media character, namely Monica Geller from the sitcom *Friends*, and its relationship to the formation of parasocial relationships—a phenomenon wherein audiences develop affective, cognitive, and behavioral connections with media characters that can influence decision making (Garcia et al., 2022a; Horton & Wohl, 1956).

Parasocial relationships are one-sided connections that audiences form with media characters, celebrities, or fictional personas. These relationships often mirror real-life social bonds, providing individuals with a sense of intimacy and interaction despite the lack of mutual engagement (Hoffner & Bond, 2022; Khairi et al., 2024; Rubin et al., 1985). Parasocial relationships have become an increasingly relevant area of study in media psychology, as they influence viewers’ attitudes, behaviors, and even decision-making processes (Bond, 2021; Giles, 2002). To capture the multidimensional nature of parasocial relationships, Garcia and colleagues (Garcia et al., 2022a) proposed the Affective, Behavioral, Cognitive, and Decisional model. This framework emphasizes four dimensions of engagement: the emotional connection viewers feel toward a character (Affective), the extent to which they emulate or quote the character in daily life (Behavioral), their intellectual investment in the character’s thoughts and actions (Cognitive), and how the character’s values influence their choices (Decisional). This multidimensional perspective allows researchers to delve deeper into how and why media characters resonate with audiences, extending beyond mere entertainment to shape social identity and interpersonal dynamics.

Parasocial relationships are deeply influenced by how audiences perceive and relate to media characters’ personality traits. Research suggests that individuals are drawn to characters who exhibit traits they find relatable, aspirational, or emotionally engaging, forming connections that mirror real-life interpersonal bonds (Giles, 2002; Möri & Fahr, 2023; Rubin et al., 1985). These connections often fulfill psychological needs such as companionship, validation, and emotional support, particularly for individuals experiencing loneliness or social isolation (Rubin et al., 1985; Stein et al., 2024). Garcia and colleagues’ multidimensional model of parasocial relationships underscores how parasocial engagement reflects not only the personality traits of the character but also the traits of the audience member (Garcia et al., 2022a). In this bidirectional interplay, viewers often gravitate toward characters who embody qualities they admire in themselves or aspire to develop. Parasocial relationships, therefore, serve as a dynamic extension of viewers’ social and psychological needs—what we like in characters, celebrities, and fictional personas is often what we see in ourselves or wish to become.

## 2. Three Personality Models: Big Five, HEXACO, and Cloninger’s Biopsychosocial Model

The Big Five, or Five-Factor Model, categorizes personality into five broad dimensions: Openness to Experience, Conscientiousness, Extraversion, Agreeableness, and Neuroticism (Benet-Martínez & John, 1998; Costa & McCrae, 1980, 1984, 1992a, 1992b; McCrae & Costa, 1987, 1999). As one of the most widely validated frameworks across cultures, the Big Five has been instrumental in explaining core aspects of personality, making it highly effective in predicting behaviors in various social and professional contexts (Barrick & Mount, 1991; Bogg & Roberts, 2004; John & Srivastava, 1999; Ozer & Benet-Martínez, 2006; Roberts et al., 2007; Roberts & Hogan, 2001; Schmit & Ryan, 1993; Soto, 2019). However, a notable challenge with the Big Five is the lack of a single standardized measure. While the NEO-Personality Inventory-Revised (NEO-PI-R) is often regarded as the primary instrument (Costa & McCrae, 1992a), a wide variety of other instruments claim to measure the same five traits. For example, the Big Five Inventory (Benet-Martínez & John, 1998), the Ten-Item Personality Inventory (Gosling et al., 2003), and the International Personality Item Pool (Goldberg, 1999) offer alternative ways of assessing the Big Five. Each instrument varies in length, focus, and scope, leading to potential inconsistencies in operationalizing and interpreting the model across studies. This variability raises questions about the comparability and generalizability of findings based on different Big Five measures (Najm, 2019). Moreover, the model has been critiqued for its limited scope in capturing other dimensions of personality (e.g., moral, ethical), as it primarily describes broad, descriptive temperament traits (Cervone, 2005; Feher & Vernon, 2021; McAdams, 2001; McAdams & Manczak, 2011) rather than addressing the underlying causes or deeper moral constructs (Ashton & Lee, 2005, 2007). These critiques have spurred the development of alternative lexical models, such as HEXACO, which aim to address these conceptual gaps and provide more nuanced insights into personality structure and behavior.

The HEXACO model expands on the Big Five by introducing Honesty–Humility as a sixth factor, alongside Emotionality, eXtraversion, Agreeableness, Conscientiousness, and Openness (Ashton et al., 2006, 2004b; Ashton & Lee, 2007; K. Lee & Ashton, 2004). Honesty-Humility encompasses traits such as sincerity, fairness, modesty, and avoidance of greed, providing a robust framework for understanding social behaviors related to morality and ethics. This addition addresses a notable gap in the Big Five by explicitly including a dimension tied to ethical conduct and prosocial tendencies (Ashton & Lee, 2005; K. Lee & Ashton, 2005). For instance, individuals high in Honesty–Humility are less likely to manipulate others for personal gain, making this dimension particularly useful in predicting moral and altruistic behaviors (Hilbig et al., 2013). Additionally, studies suggest that Honesty–Humility and other HEXACO factors exhibit significant variation across cultures, further emphasizing the model’s applicability for cross-cultural research (Ashton et al., 2006, 2004b; K. Lee & Ashton, 2008). Beyond its cross-cultural utility, the HEXACO model has also demonstrated improved predictive validity over the Big Five in areas such as workplace integrity, interpersonal trust, and prosocial decision making, highlighting its practical relevance for understanding personality in diverse contexts (Ashton & Lee, 2007, 2020).

The general critique of lexical models like the Big Five and HEXACO lies in their reliance on factor-analytic approaches, which focus primarily on describing between-person differences but fall short in explaining within-person processes and the developmental aspects of personality. Factor analysis, while powerful for identifying broad trait dimensions, has been criticized for lacking a solid theoretical foundation in biological and psychological mechanisms (Anglim & O’connor, 2019; Block, 1995; Cargill, 2019; Epstein, 2010; Gould, 1981; Proulx & Morey, 2021). This limitation often results in a static view of personality, with an emphasis on temperament traits that describe automatic emotional reactions rather than the dynamic, evolving aspects of personality that reflect values, goals, and conscious self-regulation (Cervone, 2005; Garcia et al., 2019; McAbee & Connelly, 2016; McAdams, 2001; McAdams et al., 2004; McAdams & Manczak, 2011; McAdams & McLean, 2013).

By contrast, Cloninger’s Biopsychosocial Model of personality takes a more integrative approach, addressing these gaps through a dual framework of temperament and character (Ando et al., 2004; C. R. Cloninger et al., 1993, 2019; C. R. Cloninger & Zwir, 2018; K. M. Cloninger & Cloninger, 2020; del Val et al., 2024; Garcia, 2024; Garcia et al., 2024b; Gillespie et al., 2003; S. J. Lee et al., 2024; Moreira et al., 2022; Zwir et al., 2020a, 2020b, 2021, 2022). Temperament traits such as Novelty Seeking, Harm Avoidance, Reward Dependence, and Persistence are linked to heritable biological processes and unconscious emotional responses. Meanwhile, character traits such as Self-Directedness, Cooperativeness, and Self-Transcendence capture socially influenced, self-regulated goals and values, reflecting how individuals intentionally shape their identities and pursue fulfillment in life (C. R. Cloninger, 2004, 2006, 2009; C. R. Cloninger & Cloninger, 2011a, 2011b; Garcia et al., 2023). This distinction between temperament and character allows the Biopsychosocial Model to explain not only how people differ but also why they behave as they do, offering insights into personality development over the lifespan (Cervone, 2005; Zwir et al., 2021).

Cloninger’s model has demonstrated superior predictive validity compared to lexical models, particularly in areas such as well-being, resilience, and social functioning. For instance, traits like Self-Directedness and Cooperativeness are consistently associated with positive mental health outcomes, while Harm Avoidance is linked to vulnerability to stress and anxiety (Chae et al., 2019, 2020; C. R. Cloninger et al., 2010, 2012; C. R. Cloninger & Zohar, 2011; Eley et al., 2013, 2016; Garcia et al., 2022b; Grucza & Goldberg, 2007; S. J. Lee et al., 2014, 2023; Mihailovic et al., 2022; Zohar et al., 2013). Moreover, the model’s neurobiological underpinnings provide a deeper understanding of the mechanisms driving personality, linking specific traits to neurotransmitter systems, brain networks, and forms of learning (C. R. Cloninger & Zwir, 2022). By emphasizing the dynamic interplay between biology, psychology, and social influences, the Biopsychosocial Model represents a comprehensive framework for understanding personality as a whole system rather than merely a collection of traits (Cervone, 2023; McAdams & Manczak, 2011). This integrative approach is particularly valuable for studying complex phenomena such as parasocial relationships, where both automatic emotional reactions and intentional cognitive processes are at play.

## 3. The Present Study

This study aims to integrate the Big Five, HEXACO, and Biopsychosocial models to explore how audiences perceive Monica Geller’s personality and how these perceptions influence parasocial relationships with her character. Monica Geller, a central figure in the popular sitcom *Friends*, is known for her strong personality traits, such as her competitiveness, conscientiousness, and emotional intensity, making her an ideal subject for examining the interplay between perceived personality and parasocial engagement. Table 1 provides an overview of these three models and their key constructs, highlighting how they offer complementary perspectives on personality assessment and interpretation.

By applying these established personality models, this study seeks to investigate how different frameworks capture Monica’s distinct personality profile and how this relates to the multidimensional nature of parasocial relationships. This multidimensional perspective enables a nuanced exploration of the ways in which Monica’s perceived traits resonate with audiences on emotional, intellectual, and behavioral levels. Moreover, this pilot study aims to bridge personality theory and media psychology, addressing gaps in the literature regarding how personality traits of fictional characters shape audience engagement and influence viewers’ social and psychological experiences.

Ultimately, this pilot study contributes to advancing the understanding of media characters’ psychological impact on audiences, particularly in fostering meaningful yet one-sided relationships. By examining Monica Geller’s character through these theoretical lenses, the pilot study not only provides insights into the role of personality in parasocial relationships but also into the broader applicability of personality models in media contexts.

## 4. Method

### 4.1. Ethical Statement

This study was conducted in compliance with the ethical standards outlined in the 1964 Helsinki Declaration and its later amendments. Ethical board review was not required since the study involved adult participants, and no identifiable information was collected, ensuring the privacy and anonymity of all participants throughout the research process. Participants were informed about the purpose of the study, which aimed to explore personality and engagement with fictional media characters. They were made aware that their participation was voluntary and that they could withdraw from the study at any time without providing a reason. By proceeding with the survey, participants indicated their understanding of the terms of participation and provided their consent for their answers to be included in the study.

### 4.2. Participants

A total of 63 university students participated in this pilot study. The pilot study was conducted at a university known for its focus on interdisciplinary education and research in fields such as social sciences, business, and technology. The university attracts a diverse student body, including a significant proportion of international students, reflecting both local and global perspectives. The participants had an average age of 28.46 years (*SD* = 11.02) and were predominantly female (79% females, 21% males). The sample was highly educated, with 72% having completed at least an undergraduate degree. Among participants, 23.80% reported never having seen an episode of *Friends*, while 20.60% reported having seen a few episodes, but not having watched regularly. The remaining 55.50% reported that they watched several episodes (9.50%), multiple seasons (9.50%), or every season (36.50%) of the show. This distribution indicates that a majority of participants had viewed the show frequently enough to recognize Monica Geller as a central character. In addition to viewership, participants were asked to rate their familiarity with Monica Geller. A total of 14.30% reported no familiarity with the character, 30.20% reported basic familiarity, 12.70% reported moderate familiarity, 15.30% reported being very familiar, and 17.50% described themselves as extremely familiar with the character. These measures ensured that participants had varying levels of exposure and familiarity, providing a range of perspectives for analyzing perceptions of Monica Geller’s personality and parasocial engagement.

For contextual comparison, normative population data were drawn from prior studies that evaluated personality traits across large, representative samples. For the Big Five model, normative data came from a U.S. sample of 711 individuals (Benet-Martínez & John, 1998). Norms for the HEXACO model were derived from a U.S. sample of 1126 individuals, as reported on the HEXACO website (https://hexaco.org). For Cloninger’s Biopsychosocial Model, normative data were obtained from a U.S. sample of 1948 individuals, as documented by the Anthropedia Foundation (https://anthropedia.org). These datasets served as benchmarks for calculating *Z*-scores, enabling comparisons between participants’ perceptions of Monica Geller’s personality traits and broader population norms.

### 4.3. Procedure

The study was conducted online using an anonymous survey designed to evaluate participants’ perceptions of Monica Geller’s personality through the lens of the three personality models (Big Five, HEXACO, and Biopsychosocial) and their parasocial relationship with her character. The survey included several sections: participants’ demographic information, their viewership for *Friends*, familiarity with Monica Geller, parasocial relationship engagement, and personality assessments.

To provide a shared context for evaluating Monica Geller, the survey incorporated a brief (eight minutes) embedded video compilation titled “Monica Geller’s Best Moments”. The video showcased a curated selection of scenes highlighting Monica’s defining traits, including her competitiveness, organizational skills, emotional expressiveness, and humor. The video was presented after participants completed questions on demographics, viewership, familiarity, and parasocial engagement, but prior to responding to the personality instruments. This ensured a consistent and vivid reference point for participants when assessing Monica Geller’s personality. To mitigate order effects and potential biases, the order in which participants encountered the personality instruments was randomized.

Participants were explicitly instructed to respond to all survey items based on their perceptions of Monica Geller as portrayed in the sitcom *Friends*. By utilizing an online platform and integrating multimedia stimuli, the study ensured accessibility, anonymity, and a controlled presentation of the target character. This design facilitated a focused and standardized analysis of personality perceptions and parasocial relationships.

### 4.4. Measures

#### 4.4.1. Viewership and Familiarity

Viewership of *Friends* was measured with the question “How much have you watched the sitcom *Friends*?” (1 = *Never watched—I have never seen an episode*, 5 = Watched extensively—*I’ve watched every season or most episodes multiple times*). Familiarity with the character, Monica Geller, was measured with the question “How familiar are you with the character ’Monica Geller’ from *Friends*?” (1 = *Not familiar—I do not know the character*, 5 = Extremely familiar—*I know the character in great detail*, *including most of her personality traits*, *relationships*, *and major story arcs*). The responses to these two questions were highly correlated (*r* = 0.942, *p* < 0.001), which was expected since increased viewership should lead to higher familiarity with the characters in the sitcom.

#### 4.4.2. Personality

Participants’ perceptions of Monica Geller’s personality were assessed using a modified version of three established personality instruments: the Big Five Inventory (BFI, 44 items) (Benet-Martínez & John, 1998), the HEXACO-60 (60 items) (Ashton & Lee, 2009), and the Temperament and Character Inventory Revised (TCI-R III 60, 60 items) (Garcia et al., 2024a). Participants rated Monica Geller on a 5-point Likert scale (1 = *Strongly Disagree*, 5 = *Strongly Agree*) across the Big Five dimensions: Openness (e.g., “Monica is curious about different things”), Conscientiousness (e.g., “Monica does a thorough job”), Extraversion (e.g., “Monica is talkative”), Agreeableness (e.g., “Monica is helpful and unselfish with others”), and Neuroticism (e.g., “Monica gets depressed, blue”); the HEXACO dimensions: Honesty–Humility (e.g., “Monica wouldn’t use flattery to get a raise or promotion at work, even if she thought it would succeed.”), Emotionality (e.g., “Monica sometimes can’t help worrying about little things.”), Extraversion (e.g., “Monica prefer jobs that involve active social interaction to those that involve working alone.”), Agreeableness (e.g., “Monica rarely holds a grudge, even against people who have badly wronged her.”), Conscientiousness (e.g., “Monica often push herself very hard when trying to achieve a goal.”), and Openness (e.g., “Monica would enjoy creating a work of art, such as a novel, a song, or a painting.”); and the TCI-dimensions: Novelty Seeking (e.g., “Monica often tries new things just for fun or thrills, even if most people think it is a waste of time.”), Harm Avoidance (e.g., “Monica often feels tense and worried in unfamiliar situations, even when others feel there is little to worry about.”), Reward Dependence (e.g., “Monica likes to discuss her experiences and feelings openly with friends instead of keeping them to herself.”), Persistence (e.g., “The harder a job is the more Monica likes it.”), Self-Directedness (e.g., “Repeated practice has given Monica good habits that are stronger than most momentary impulses or persuasion.”), Cooperativeness (e.g., “Monica can usually accept other people as they are, even when they are very different from her.”), and Self-Transcendence (e.g., “Monica has had moments of great joy in which she suddenly had a clear, deep feeling of oneness with all that exists.”).

### 4.5. Engagement with Parasocial Relationships

A modified version of the Multidimensional Measure of Parasocial Relationships (MMPR) (Garcia et al., 2022a) was used to assess participants’ parasocial engagement with Monica Geller. The MMPR consists of 18 items rated on a 5-point Likert scale (1 = *Strongly Disagree*, 5 = *Strongly Agree*) that measures four dimensions: Affective (e.g., “I experience that I get emotionally engaged when I see Monica in the *Friends* episodes.”), Behavioral (e.g., “I often quote Monica when talking to my friends or when posting is social media.”), Cognitive (e.g., “Monica seems to be a genuine person that, is she was real, I would get along with in real life.”), and Decisional (e.g., “I prefer things that Monica uses or talk about in the *Friends* episodes (e.g., products, nutrition advice, training advice, etc.) before similar things by other characters.”).

### 4.6. Data Analysis

All analyses were conducted using SPSS Version 29.0. Effect sizes were reported where relevant, providing additional context for interpreting the strength and practical significance of the findings. Descriptive statistics were calculated to summarize participants’ ratings of Monica Geller across all personality traits from the Big Five, HEXACO, and Biopsychosocial models, as well as their engagement across the Affective, Behavioral, Cognitive, and Decisional dimensions of parasocial relationships. Means, standard deviations, and ranges were reported for all variables to provide an overview of the data distribution.

Additionally, participants’ perceptions of Monica Geller’s personality were contextualized by comparing their ratings to normative population data. Z-scores were calculated based on established benchmarks for each personality model. Big Five: U.S. sample (*N* = 711) from Benet-Martínez and John (1998), HEXACO: U.S. sample (*N* = 1126) from the HEXACO Personality Inventory database (https://hexaco.org), and Biopsychosocial Model: U.S. sample (*N* = 1948) from the Temperament and Character Inventory database (https://anthropedia.org). These standardized scores allowed for the evaluation of deviations in Monica Geller’s perceived personality traits relative to typical population distributions, providing insights into how Monica’s personality profile diverges from real-world norms. The comparisons aimed to contextualize how participants’ perceptions of Monica may reflect or amplify distinctive aspects of her character portrayal in *Friends*.

Pearson correlation analyses were conducted to explore the relationships between participants’ perceptions of Monica Geller’s personality traits and their levels of parasocial engagement. These analyses examined whether specific personality traits attributed to Monica were associated with higher engagement across the four dimensions of the Affective, Behavioral, Cognitive, and Decisional model.

## 5. Results

### 5.1. Personality Ratings

#### 5.1.1. Big Five

**Figure 1:**
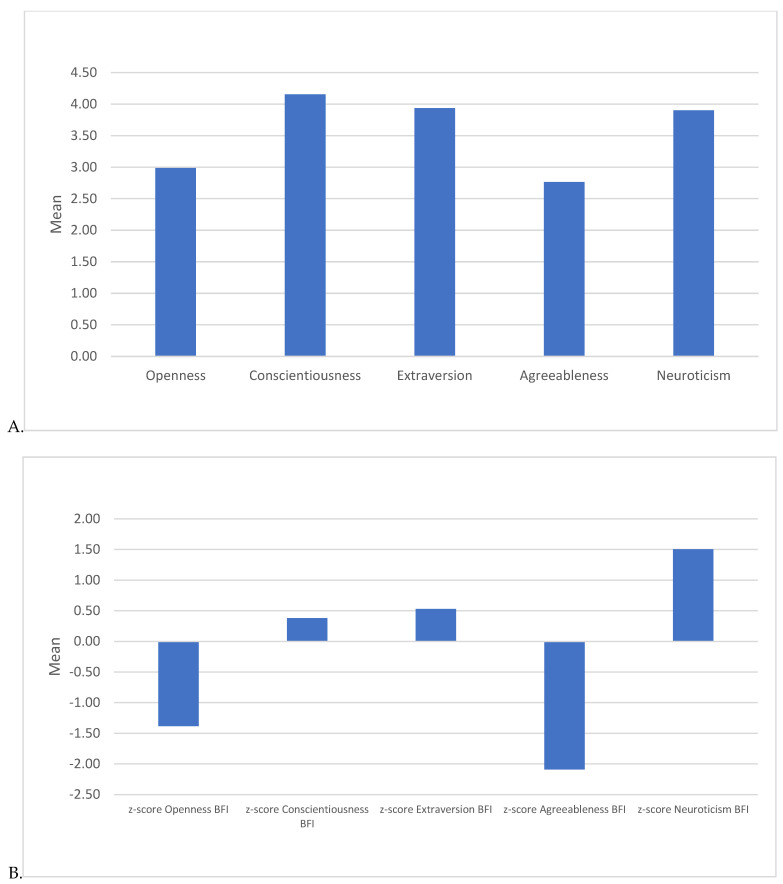
Means in (**A**) raw scores and (**B**) *Z*-scores (based on norm US-data) for participants’ perception of Monica Geller’s personality as measured by the Big Five Inventory (BFI).

Monica was rated highest on Conscientiousness (*M* = 4.15, *SD* = 0.55), reflecting her meticulous, organized nature. Her Agreeableness was rated lowest (*M* = 2.77, *SD* = 0.63), suggesting her being perceived as having less supportive and caring interactions with other characters. Extraversion scored moderately high (*M* = 3.93, *SD* = 0.47), indicating that participants perceived Monica as sociable. Neuroticism was moderate high (*M* = 3.90, *SD* = 0.38), corresponding to her occasional anxiety-driven behaviors. Her Openness score (*M* = 2.99, *SD* = 0.55) was lower, suggesting a character more focused on familiarity and routine than novelty and variety. See Figure 1.

To further contextualize Monica’s Big Five ratings, *Z*-scores were calculated based on the U.S. population norms. The *Z*-score for Openness was −1.38, suggesting that Monica is viewed as significantly less open to new experiences, underscoring her more rigid and traditional preferences. With a *Z*-score of +0.38, Monica’s Conscientiousness level is slightly above average, reflecting her portrayal as highly organized and responsible. Monica’s Extraversion score yielded a *Z*-score of +0.53, indicating she is perceived as somewhat more sociable than the average person, aligning with her active social engagement. Her Agreeableness score produced a *Z*-score of −2.09, suggesting a significantly lower-than-average score in Agreeableness. This underscores her competitive and, at times, less empathetic tendencies, aligning with aspects of her character that are direct and occasionally confrontational. With a *Z*-score of +1.50, Monica’s Neuroticism score is notably above average, reflecting her tendency toward anxiety and emotional intensity, particularly in high-stress scenarios. These results indicate that Monica Geller’s personality profile, as perceived by viewers, deviates notably from average population norms in Agreeableness, Neuroticism, and Openness. She is perceived as highly conscientious and sociable, but less cooperative and flexible, reinforcing her character’s unique personality traits as portrayed in *Friends*. See Figure 1.

#### 5.1.2. HEXACO

**Figure 2:**
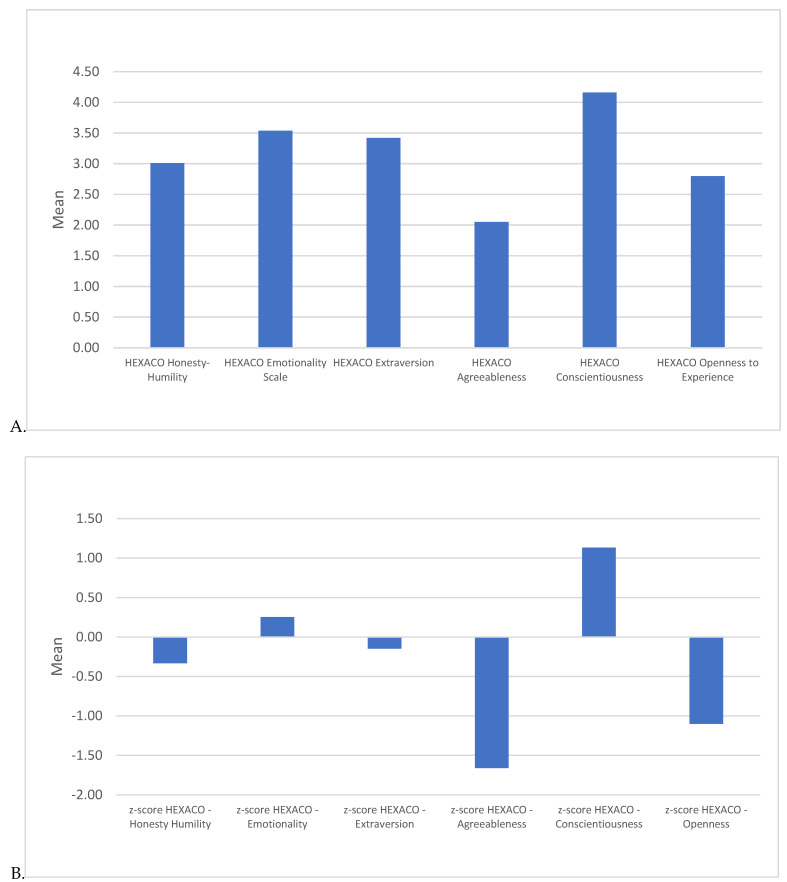
Means in (**A**) raw scores and (**B**) *Z*-scores (based on U.S. norm data) for participants’ perception of Monica Geller’s personality as measured by the HEXACO-60.

Monica’s Honesty–Humility score was moderate (*M* = 3.01, *SD* = 0.58), indicating that participants perceive her as somewhat sincere, but not exceptionally modest or self-effacing. Emotionality was moderately high (*M* = 3.54, *SD* = 0.45), aligning with Monica’s tendency toward strong emotional reactions and moments of vulnerability. Her Extraversion was moderate (*M* = 3.42, *SD* = 0.45), showing that participants view her as sociable and lively, though not as highly extroverted as other characters. Monica scored lowest on HEXACO Agreeableness (*M* = 2.05, *SD* = 0.55), suggesting that participants perceive her as less forgiving and more confrontational. Similar to the Big Five, her HEXACO Conscientiousness was high (*M* = 4.16, *SD* = 0.66), reflecting her disciplined and organized nature. Openness was low (*M* = 2.80, *SD* = 0.47), indicating a preference for familiarity over novelty. See Figure 2. To further contextualize Monica’s HEXACO ratings, *Z*-scores were calculated against the U.S. population norms. The *Z*-score for Honesty–Humility was −0.33, indicating Monica is slightly less modest and sincere than the average person. Emotionality yielded a *Z*-score of +0.25, suggesting a slightly higher emotional intensity. Monica’s Extraversion *Z*-score was −0.15, showing a slightly lower sociability compared to typical norms. With a *Z*-score of −1.66, her low Agreeableness rating aligns with her direct, sometimes confrontational style. Her Conscientiousness *Z*-score of +1.13 shows a significant inclination toward discipline and organization. Monica’s Openness *Z*-score of −1.10 reflects a marked preference for routine and predictability over novelty. These results underscore Monica’s unique character, reflecting high Conscientiousness and moderate Emotionality but notably low Agreeableness and Openness. These traits contribute to her structured, emotionally expressive, and at times, inflexible personality as perceived by viewers. See Figure 2.

#### 5.1.3. Cloninger’s Biopsychosocial Model

**Figure 3:**
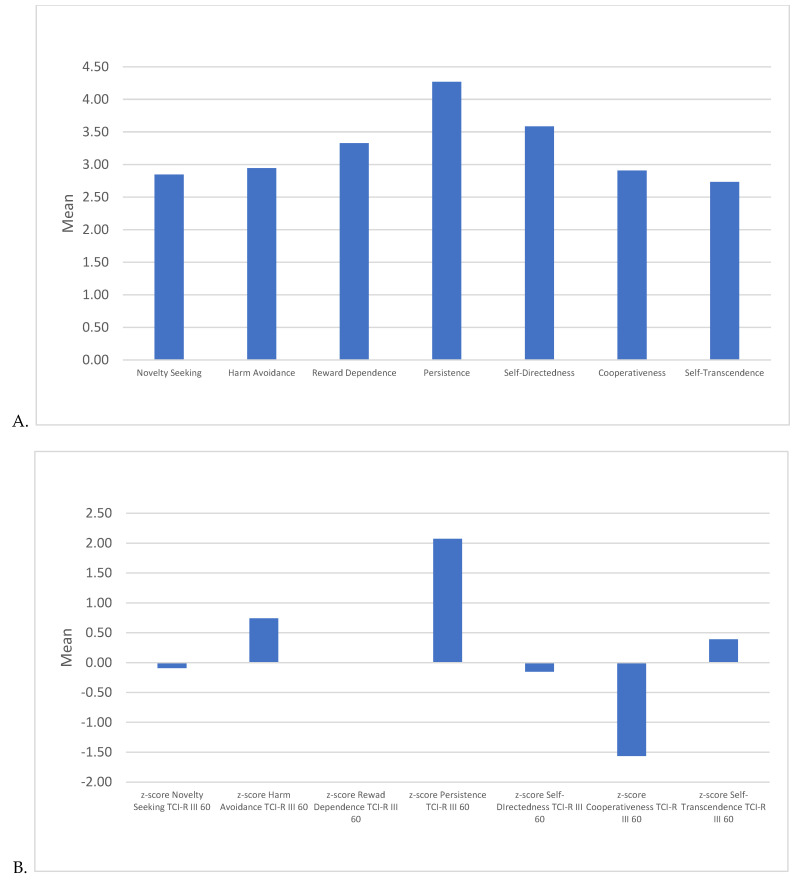
Means in (**A**) raw scores and (**B**) *Z*-scores (based on U.S. norm data) for participants’ perception of Monica Geller’s personality as measured by the Temperament and Character Inventory Revised III (TCI-R III 60).

Monica’s Novelty Seeking score was average (*M* = 2.85, *SD* = 0.71), indicating that participants perceived her as not particularly drawn to new or exciting experiences nor having a preference for familiarity and routine. Harm Avoidance was also moderate (*M* = 2.95, *SD* = 0.51), aligning with her cautious and sometimes anxious approach to situations that may involve risk or uncertainty and sometimes being optimistic and calm. Reward Dependence was moderately high (*M* = 3.33, *SD* = 0.57), showing that Monica is seen as socially oriented and approval-seeking, valuing positive reinforcement from others. Monica scored highest on Persistence (*M* = 4.27, *SD* = 0.56), reflecting her driven, determined, and perfectionistic nature and her tendency to persistently pursue goals even in the face of obstacles. Her Self-Directedness score was moderately high (*M* = 3.59, *SD* = 0.48), suggesting that she is seen as somewhat self-regulated and responsible, though not exceedingly so. Cooperativeness was low (*M* = 2.91, *SD* = 0.68), indicating that Monica may be perceived as less accepting and forgiving, aligning with her competitive and assertive tendencies. Monica’s score on Self-Transcendence was the lowest (*M* = 2.73, *SD* = 0.53), suggesting a more practical, grounded approach, with less inclination towards abstract or spiritual thinking. See Figure 3.

To contextualize Monica’s ratings on the TCI dimensions, *Z*-scores were calculated based on the U.S. population norms. With a *Z*-score of −0.09, Monica’s Novelty Seeking is close to average, indicating a slight preference for familiarity over novelty but that she can also be perceived in the opposite way in other contexts. Harm Avoidance yielded a *Z*-score of +0.74, suggesting that Monica is somewhat more cautious and risk-averse than the average person. The *Z*-score for Reward Dependence was close to zero (−0.002), indicating that Monica’s orientation towards social approval and reinforcement is average. That is, she can be perceived as objective and independent in certain situations and be perceived as dependent, afraid of conflicts, and warm in others. Persistence had the highest *Z*-score at +2.07, underscoring Monica’s notably above-average determination, perfectionism, and hard-working attitude. With a *Z*-score of −0.15, Monica’s Self-Directedness is slightly below average, showing that while she is relatively responsible, she may not be as self-acceptant and self-determined as other people. Her low Cooperativeness yielded a *Z*-score of −1.56, supporting the perception that Monica is less forgiving and more competitive. Monica’s Self-Transcendence *Z*-score was +0.39, indicating a modestly average inclination towards abstract thinking, though she remains pragmatic. These results highlight Monica Geller’s unique personality traits as perceived by viewers, with high Persistence and moderate Reward Dependence but notably low Cooperativeness and Self-Transcendence. This combination reinforces her character’s driven, goal-oriented focus and her occasional lack of flexibility and empathy, which are consistent with her portrayal on *Friends*. See Figure 3.

### 5.2. Parasocial Relationship Engagement

**Figure 4:**
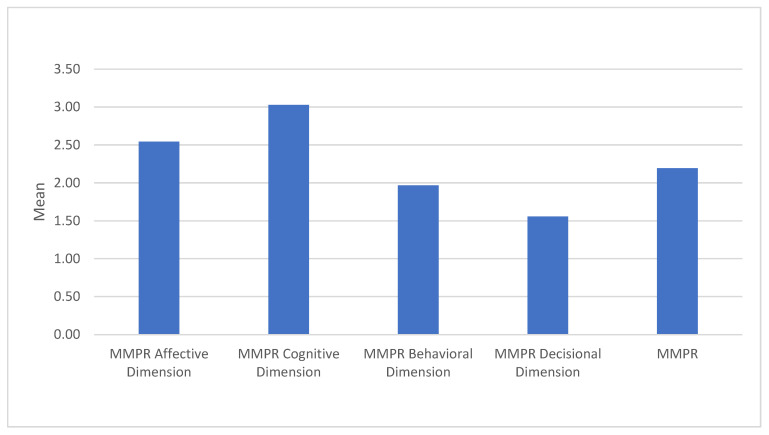
Means for each parasocial relationship dimension, as measured by the Multidimensional Measure of Parasocial Relationships (MMPR), illustrating the extent of affective, cognitive, behavioral, and decisional engagement with Monica Geller.

Participants rated their emotional connection with Monica moderately (*M* = 2.54, *SD* = 0.52), suggesting that viewers feel a modest emotional bond with her (Affective Dimension). The Cognitive dimension was the highest (*M* = 3.03, *SD* = 0.56), indicating that participants think about Monica and relate her actions and values to their own lives in a moderate way. Engagement in the Behavioral dimension was lower (*M* = 1.97, *SD* = 0.57), showing that participants are less likely to imitate or act based on Monica’s behaviors, reflecting a more passive engagement style. The Decisional dimension received the lowest score (*M* = 1.56, *SD* = 0.46), indicating that Monica has minimal influence on participants’ personal decisions or choices, consistent with viewers’ understanding of her as a fictional character. These results reveal that Monica Geller elicits a stronger cognitive engagement from viewers, with moderate emotional connections, but limited behavioral influence and minimal decisional impact. This pattern suggests that while Monica resonates intellectually and emotionally, viewers maintain clear boundaries between her fictional character and their personal lives. See Figure 4.

### 5.3. Relationship Between Personality and Parasocial Relationship Engagement

The first correlation analysis was between Monica Geller’s Big Five personality traits and participants’ parasocial engagement with her. There was a significant positive correlation between Agreeableness and the Affective dimension (*r* = 0.259, *p* < 0.05), indicating that participants who see Monica as more agreeable tend to feel a stronger emotional connection with her character. Agreeableness also showed a significant positive correlation with Behavioral engagement (*r* = 0.384, *p* < 0.01), meaning that viewers who see Monica as more agreeable are more likely to adopt behaviors inspired by her character. Agreeableness was also significantly correlated with Cognitive engagement (*r* = 0.415, *p* < 0.01), suggesting that viewers who view Monica as more agreeable are more likely to think about her and relate her actions to their own lives. Last, a positive correlation was also found between the Decisional dimension and both Agreeableness (*r* = 0.281, *p* < 0.05) and Openness (*r* = 0.305, *p* < 0.05), indicating that viewers who perceive Monica as more kind, friendly, and open to new experiences may find her values influencing their personal decisions. The total score of the MMPR was also related to both Agreeableness (*r* = 0.456, *p* < 0.01) and Openness (*r* = 0.293, *p* < 0.05), but no other Big Five trait was significantly related to the parasocial relationship dimensions. In sum, the Big Five results suggest that viewers’ perceptions of Monica’s Agreeableness are significantly associated with all dimensions of parasocial engagement, particularly the cognitive and behavioral dimensions.

The second correlation analysis examined the relationship between Monica Geller’s HEXACO personality traits and participants’ parasocial engagement with her. A significant positive correlation was found between Honesty–Humility and the Cognitive dimension of engagement (*r* = 0.272, *p* < 0.05), indicating that participants who perceive Monica as more honest and humble are more likely to think about her character and relate her actions to their own lives. Emotionality showed a significant positive correlation with the Affective dimension (*r* = 0.320, *p* < 0.05), suggesting that viewers who see Monica as more emotionally expressive tend to feel a stronger emotional connection with her character. Additionally, Emotionality was significantly correlated with the Behavioral dimension (*r* = 0.249, *p* < 0.05), meaning that viewers who perceive Monica as more emotionally sensitive are more likely to adopt behaviors inspired by her character. Agreeableness demonstrated a positive correlation with the Decisional dimension (*r* = 0.276, *p* < 0.05), suggesting that participants who see Monica as more kind and friendly may be influenced by her values and choices in their own decision making. Both Honesty–Humility (*r* = 0.263, *p* < 0.05) and Emotionality (*r* = 0.299, *p* < 0.05) were also related to the total score of the MMPR, but no other HEXACO trait was significantly related to the parasocial relationship dimensions. In summary, the HEXACO results suggest that viewers’ perceptions of Monica’s Honesty–Humility, Emotionality, and Agreeableness are significantly associated with different dimensions of parasocial engagement. Honesty–Humility is particularly associated with cognitive engagement, Emotionality with affective and behavioral engagement, and Agreeableness with decisional engagement, reflecting a nuanced pattern in how different personality traits relate to viewer interactions with her character.

The third correlation analysis explored the relationship between Monica Geller’s TCI traits and participants’ parasocial engagement with her. Reward Dependence showed a significant positive correlation with the Cognitive dimension (*r* = 0.318, *p* < 0.05), indicating that participants who view Monica as warm, socially oriented and responsive to social reinforcement are more likely to think about her and relate to her actions. Persistence had a significant positive correlation with affective engagement (*r* = 0.310, *p* < 0.05), implying that viewers who perceive Monica as highly determined feel a stronger emotional bond with her character. Self-Directedness was positively correlated with both the Cognitive (*r* = 0.328, *p* < 0.01) and Behavioral (*r* = 0.271, *p* < 0.05) dimensions, indicating that participants who see Monica as more self-disciplined are more likely to engage cognitively and behaviorally with her character. Cooperativeness was significantly correlated with both Affective (*r* = 0.270, *p* < 0.05) and Cognitive (*r* = 0.360, *p* < 0.01) engagement, suggesting that viewers who view Monica as more cooperative and socially attuned tend to feel a stronger emotional connection and are more likely to think about her character. Additionally, Reward Dependence (*r* = 0.313, *p* < 0.05) and Cooperativeness (*r* = 0.328, *p* < 0.01) were positively associated with the overall MMPR score, suggesting a general influence on parasocial engagement when Monica was perceived as warm and sympathetic (i.e., high Reward Dependence) and tolerant and empathetic (i.e., high Cooperativeness). No other TCI trait was significantly related to the parasocial relationship dimensions. In summary, the TCI results indicate that Reward Dependence, Persistence, Self-Directedness, and Cooperativeness are associated with various dimensions of parasocial engagement with Monica Geller. Reward Dependence is particularly associated with cognitive engagement and overall parasocial connection, while Persistence enhances affective engagement. Self-Directedness and Cooperativeness contribute to both Cognitive and Affective dimensions, reflecting how Monica’s character traits influence viewers’ emotional, cognitive, and behavioral interactions with her.

## 6. Discussion

This pilot study examined perceptions of Monica Geller’s personality using three established personality models, the Big Five, HEXACO, and Cloninger’s Biopsychosocial Model, and their relationship to parasocial engagement. The findings highlight how Monica’s perceived traits, such as high Conscientiousness and low Agreeableness, align with her depiction as a driven and competitive character in *Friends*. Across the three models, traits linked to social interaction and emotional responsiveness, including Honesty–Humility, Reward Dependence, and Cooperativeness, demonstrated significant correlations with parasocial relationship dimensions. Notably, the TCI model offered the most comprehensive coverage of Monica’s personality traits in relation to the cognitive, affective, and overall parasocial engagement. These results underscore the dynamic role of personality perceptions in shaping viewers’ one-sided relationships with fictional characters.

The strong association of Agreeableness (Big Five) and Emotionality (HEXACO) with Affective engagement underscores the importance of warmth, empathy, and emotional responsiveness in fostering emotional bonds with media characters. These traits appear central to viewers forming meaningful connections, reflecting Horton and Wohl’s (Horton & Wohl, 1956) foundational conceptualization of parasocial relationships as emotionally supportive and pseudo-social in nature (see also Giles, 2002; Hartmann et al., 2008; Rubin et al., 1985). Characters who are perceived as agreeable and emotionally expressive provide audiences with a sense of companionship and understanding, fulfilling psychological needs such as validation and belonging. This alignment between perceived personality traits and affective engagement holds significant implications for media psychology. Specifically, it suggests that characters portrayed with high levels of agreeableness and emotional depth are more likely to evoke lasting emotional bonds, enhancing audience retention and loyalty (Rubin et al., 1985) These findings further highlight the potential for carefully crafted character traits to influence not only individual viewer experiences but also broader media consumption patterns.

The Biopsychosocial model, with its dual emphasis on both temperament (i.e., biologically driven automatic emotional responses) and character (i.e., goals and values in relation to the self, others and one’s existence), was expected to offer an integrative framework for understanding how personality traits shape parasocial engagement. Unlike lexical models, which focus on descriptive traits (McAdams, 2008; McAdams & Manczak, 2011), this model provides a dynamic and integrative perspective that captures the complexity of personality (Cervone, 2005; C. R. Cloninger, 2004), even when assessing fictional characters such as Monica Geller. This dynamic and integrative approach facilitates a deeper understanding of how distinct personality traits resonate with viewers and elucidates the multifaceted ways they engage with media characters. For instance, the temperament trait of Reward Dependence and the character trait of Cooperativeness, while both addressing interpersonal connections, represent distinct dimensions within the Biopsychosocial model. High Reward Dependence reflects tendencies toward sentimentality, a readiness for warm communication, and approval-seeking behaviors. In contrast, high Cooperativeness encompasses tolerance, empathy, helpfulness, forgiveness, and principled behavior, demonstrating how socially developed character traits differ from biologically rooted temperamental tendencies and motivations (Garcia et al., 2014, 2017; Mousavi et al., 2015).

In this study, perceiving Monica as high in Reward Dependence was significantly correlated with Cognitive engagement, indicating that viewers who perceive Monica as sentimental, attention-seeking, and open to warm communication are more likely to form an intellectual and evaluative bond with her. This bond is characterized by admiration for her values, authenticity, and positive qualities, as well as a perceived sense of compatibility in hypothetical real-life interactions (i.e., high Cognitive engagement). In contrast, perceiving Monica as empathetic and socially tolerant (i.e., high Cooperativeness) was associated with heightened Affective engagement, fostering an emotionally resonant, engaging, and personally meaningful parasocial relationship. These findings underscore the nuanced ways in how personality traits influence distinct dimensions of parasocial relationships; traits promoting social connectedness and warmth emerge as crucial in shaping how audiences perceive and relate to fictional characters, albeit influencing specific aspects of parasocial relationship engagement differently. Moreover, the temperament trait of Persistence and the character trait of Cooperativeness were strongly associated with Affective engagement. This suggests that Monica’s determination, industriousness, perfectionism and her empathetic and helpful nature enhance viewers’ emotional bonds with her. Hence, characters who exhibit strong willpower and cooperative tendencies are likely to evoke admiration and emotional resonance, making them more relatable and impactful. The character trait of Self-Directedness emerged as the sole predictor of Behavioral engagement, indicating that viewers are more likely to emulate or discuss characters they perceive as self-accepting, responsible, and resourceful. In other words, characters who embody self-determination inspire viewers to act upon or integrate their behaviors and traits into their own lives. Additionally, the temperament trait of Reward Dependence, alongside the character traits of Cooperativeness and Self-Directedness, were significantly associated with Cognitive engagement and overall parasocial engagement. Monica’s perceived warmth, approval-seeking behavior, kindness, and self-determined personality appear to facilitate intellectual connections, encouraging viewers to think critically about her actions and values and relate them to their own lives. These findings underscore the Biopsychosocial model’s robust capacity to capture the multidimensional nature of parasocial engagement, effectively illustrating how diverse personality dimensions influence audience–character connections. This has significant implications for personality psychology, emphasizing the value and importance of integrating both temperament and character traits to comprehensively understand the complex underpinnings of viewer–character relationships.

In sum, this pilot study highlights the unique contributions of the Biopsychosocial Model, alongside the Big Five and HEXACO frameworks, in advancing our understanding of parasocial relationships. The Biopsychosocial Model provided the most comprehensive perspective, capturing the interplay of temperament and character traits across Affective, Behavioral, Cognitive, and overall parasocial engagement dimensions. In contrast, the Big Five and HEXACO models offered valuable insights into the Decisional dimension of parasocial engagement. Traits like Agreeableness (Big Five) and Honesty–Humility (HEXACO) were particularly associated with viewers’ alignment with Monica’s values and choices, suggesting that personality traits fostering trustworthiness, moral alignment, and social adaptability influence viewers’ willingness to emulate or adopt the behaviors and preferences of media characters. Together, these models provide a nuanced, multidimensional understanding of how personality traits drive distinct facets of parasocial relationships, underscoring the value of integrating diverse theoretical frameworks in media psychology research.

### 6.1. Limitations and Strengths

As any other pilot, there are several limitations related to methodology, model comparisons, and the specificity of the sitcom character analyzed, that should be considered when interpreting the findings. First, the sample size was small and limited to university students, which constrains the generalizability of the results to broader populations. However, this pilot study incorporated normative data from larger samples (*N*_BFI_ = 711, *N*_HEXACO_ = 1126, *N*_TCI_ = 1948) to contextualize participants’ perceptions of Monica’s personality within each model, enhancing the interpretability of the findings despite the sample limitations. That being said, the study’s cross-sectional design limits the ability to infer causal relationships between personality perceptions and parasocial engagement. Longitudinal studies are needed to explore how personality-driven engagement evolves over time and whether viewers’ perceptions of character traits change with repeated exposure. The reliance on inferential statistics in a small, non-representative sample also raises concerns about statistical power, validity, and reliability. While significant correlations were identified, these results should be interpreted cautiously, as they may not generalize to broader populations despite the use of normative data for contextualizing participants’ assessment of Monica Geller’s personality. The small sample size may have also increased the likelihood of Type I or Type II errors, further emphasizing the exploratory nature of this pilot study. Future research should prioritize larger and more diverse samples to enable more advanced statistical analyses (e.g., mediation and/or moderation), enhance statistical power, and provide more reliable effect size estimates. Such efforts would allow more robust conclusions about the relationship between personality traits and parasocial engagement and determine whether these findings hold across different demographics, cultural contexts, and levels of familiarity with the sitcom *Friends* and its characters.

Second, differences in the operationalization of similar traits across the personality models posed challenges for direct comparisons. For instance, while Big Five Agreeableness demonstrated consistent and significant correlations with nearly all dimensions of parasocial engagement, analogous traits such as HEXACO Agreeableness and TCI Reward Dependence and Cooperativeness showed less consistent associations. This discrepancy likely stems from differences in the conceptual scope of these traits. Big Five Agreeableness broadly captures warmth, friendliness, and cooperativeness—qualities that align closely with the general expectations of supportive parasocial relationships. In contrast, HEXACO Agreeableness places greater emphasis on forgiveness and tolerance, traits that may not resonate as strongly with Monica Geller’s character or the dimensions of parasocial engagement assessed in this study. The Biopsychosocial Model’s Cooperativeness extends beyond temperament-based traits to include socially developed values like empathy, helpfulness, and principled behavior. These conceptual variations highlight the unique focus of each model in capturing specific facets of personality. For example, Monica Geller’s driven and competitive nature may align less with HEXACO Agreeableness, which emphasizes forgiveness, but more with Big Five Agreeableness, which includes cooperative tendencies. Similarly, the Biopsychosocial Model’s Cooperativeness provided insights into Monica’s socially principled behavior, which aligns with the affective dimension of parasocial engagement. Notably, the Biopsychosocial Model captured these nuances through Reward Dependence and Cooperativeness, which were linked to distinct parasocial relationship dimensions, highlighting the model’s strength in differentiating between interpersonal traits that correspond to distinct dimensions of personality. Likewise, while HEXACO Emotionality demonstrated stronger associations with affective and behavioral engagement compared to Big Five Neuroticism, this probably reflects HEXACO’s broader definition of Emotionality, encompassing emotional sensitivity and vulnerability. This nuance in trait definitions highlights that even traits with conceptual similarities can capture distinct aspects of personality that influence parasocial engagement differently, depending on the emphasis within each model. That being said, these issues might also stem from the use of short measures, which can limit the depth and precision of personality trait assessments across models (Gosling et al., 2003; Guerra et al., 2024).

Third, while Monica was chosen as a well-known and multidimensional character for this exploratory pilot study, the focus on a single character restricts the generalizability of the findings. Monica’s unique personality traits, including her high Persistence (TCI), emotional intensity (HEXACO Emotionality), and low Agreeableness (Big Five), may naturally align more closely with certain trait dimensions in one model compared to another. For instance, while her high Persistence aligns well with affective engagement, HEXACO’s Emotionality seems to capture her vulnerability in ways that Big Five Neuroticism does not. These results may therefore be character-specific, and findings could differ for other sitcom characters with contrasting personalities or narrative roles. Future studies analyzing a wider array of characters, genres, and media types would help establish whether these patterns generalize across diverse media contexts.

Another limitation pertains to the adaptation of established personality inventories to assess perceptions of a fictional character’s traits. This approach aligns with practices in other-reports (e.g., friend or parent assessments) and was deemed necessary to capture participants’ perceptions of Monica Geller within the study’s framework. Research on other-report measures in personality assessment reveals their value and comparability to self-reports. Meta-analyses, for example, show that other-ratings can provide accurate and predictive personality assessments, particularly when raters have interpersonal familiarity or contextual knowledge of the target (Connelly & Ones, 2010). Importantly, the structure of personality remains consistent across self- and other-reports, underscoring the robustness of personality constructs (Nuzum et al., 2019). While some discrepancies in perceptions exist, especially regarding age-related changes (Rohrer et al., 2018), the overall utility of other-reports in enriching our understanding of personality is well supported, with no significant differences between self- and informant-report means in most contexts (Kim et al., 2019). These findings suggest that incorporating other-report perspectives, even for fictional characters, can yield meaningful insights. However, due to sample size limitations, the psychometric properties of the adapted measures (e.g., confirming the factor structure of the instruments) were not formally evaluated in this study, which may affect the reliability and validity of the findings. Additionally, the indirect nature of the assessment introduces potential biases, such as projection bias, where participants may attribute their own traits or values to Monica Geller. While this represents a limitation, it was anticipated due to the complex ways individuals project and relate to fictional characters within different dimensions of parasocial relationships. In essence, what draws our attention to others often mirrors what we like and dislike (i.e., our temperament) or what we value (i.e., our character). Nevertheless, future studies should prioritize validating adapted instruments for fictional characters to enhance methodological rigor and deepen our understanding of personality perception in media contexts.

Despite these limitations, the pilot study also possesses significant strengths. By integrating three established personality models, the research provides a multidimensional exploration of how personality traits shape parasocial relationships. The inclusion of the Biopsychosocial Model, with its focus on both temperament and character, offered novel insights into how specific personality traits influence distinct parasocial engagement dimensions, thus pointing towards new research venues that can help to expand the theoretical understanding of parasocial relationships. Additionally, the study leveraged standardized measures and population norms for contextual comparisons, enhancing the validity of the findings. Recognizing the inherent subjectivity in assessing personality traits, particularly for fictional characters, the study utilized a standardized video compilation of Monica Geller’s “best moments” to provide a consistent reference point across participants. While this approach cannot fully eliminate biases, such as projection bias, it serves to reduce variability in individual assessments and ensures a shared portrayal of the character, thereby bolstering the reliability of the findings. However, the reliance on self-reported data remains a limitation. Future studies could integrate more objective methods, such as behavioral coding of onscreen behavior, to complement self-reported measures and provide a more robust assessment of personality traits and their influence on parasocial engagement.

### 6.2. Implications

This pilot study offers significant implications for both academic and applied fields, particularly in media psychology, personality assessment, and education. From an academic perspective, the findings emphasize the value of integrating multiple personality frameworks to capture the multifaceted nature of parasocial relationships. While the Big Five and HEXACO models provide valuable insights into broad traits like Agreeableness and Emotionality, the Biopsychosocial model stands out for its ability to address dynamic interactions between temperament and character traits, thereby encompassing affective, cognitive, and social dimensions of parasocial engagement. This underscores the importance of employing diverse theoretical approaches when studying complex psychological phenomena, such as the ways audiences relate to media characters.

From an applied perspective, these findings are relevant for media creators and marketers. Understanding how specific personality traits of fictional characters resonate with audiences can inform the design of relatable, emotionally engaging characters that foster viewer loyalty and influence audience behaviors. Traits like Agreeableness and Honesty–Humility, which were associated with decisional engagement, provide actionable insights into how characters can be portrayed as trustworthy and morally aligned to enhance their impact on viewers.

The study also has pedagogical implications for the teaching of psychological theories and models, particularly in personality and media psychology courses as well as introductory courses. Demonstrating the unique contributions of each personality framework in real-world applications can deepen students’ understanding of the strengths and limitations of these models. For instance, while the Big Five and HEXACO highlight general descriptive traits, the Biopsychosocial model offers an integrative approach that links personality to broader emotional, cognitive, and social outcomes. Teaching students to apply these frameworks to analyze media characters can enhance their skills in personality assessment, critical thinking, and the interpretation of psychological constructs in diverse contexts. Such an approach prepares future psychologists to apply theoretical knowledge in practical, interdisciplinary settings, bridging the gap between research and real-world application.

## 7. Conclusions and Last Remarks

This study underscores the value of integrating diverse personality frameworks to understand parasocial relationships with media characters. The findings highlight the Biopsychosocial model’s capacity to provide a comprehensive perspective by offering nuanced insights into cognitive, affective, and overall parasocial engagement. By capturing the multidimensional nature of viewer–character connections, the Biopsychosocial model extends beyond the descriptive traits emphasized in lexical models to include the dynamic interplay of temperament and character. Moreover, the application of the Big Five and HEXACO models enriched the understanding of specific parasocial dimensions, such as decisional engagement, emphasizing how traits like Agreeableness and Honesty–Humility shape viewers’ alignment with a character’s values and behaviors. Together, these models illustrate the multifaceted role of personality in media psychology, providing a framework for exploring how fictional characters resonate with audiences on emotional, intellectual, and behavioral levels. By expanding the scope of personality and media psychology research, scholars can gain deeper insights into the mechanisms through which media characters shape attitudes, behaviors, and social connections in the audience.

## Figures and Tables

**Table 1:** Overview of the Big Five, HEXACO, and Biopsychosocial Models of personality and their key constructs.

Theory	Definition of Personality	Hypotheses	Model	Dimensions	Traits	Measurement	Example of Questions	Method
**N/A**	Personality consists of recurring characteristics in the form of emotions, thoughts, and behaviors that distinguish one person from others.	Relevant and prominent characteristics of the individual are encoded in language (John et al., 1988)	Big Five	Temperament	**O**penness	The Revised NEO Personality Inventory Revised (NEO-PI-R)	**O:** I have active imagination.	Factor analysis
				(+ Character to some extent, specially, prosocial traits)	**C**onscientiousness	Big Five Inventory (BFI)	**C:** I persevere until the task is finish.	
					**E**xtraversion	Ten Item Personality Measure	**E:** I am talkative.	
					**A**greeableness	Etc.	**A:** I am helpful and unselfish with others.	
					**N**euroticism		**N:** I get nervous easily.	
			HEXACO	Temperament	**H**onesty-Humility	The HEXACO Personality Inventory Revised	**H:** I wouldn’t use flattery to get a raise or promotion at work, even if I thought it would succeed.	
				(+ Character to some extent, specially, prosocial traits)	**E**motionality		**E:** When it comes to physical danger, I am very fearful.	
					E**x**traversion		**X:** I prefer jobs that involve active social interaction to those that involve working alone.	
					**A**greeableness		**A:** I am usually quite flexible in my opinions when people disagree with me.	
					**C**onscientiousness		**C:** I plan ahead and organize things, to avoid scrambling at the last minute.	
					**O**penness to Experience		**O:** People have often told me that I have a good imagination.	
**EVOLUTION**	Personality is the dynamic organization of biopsychosocial systems within an individual, through which the individual uniquely shapes and adapts to an ever-changing internal and external environment.	A person’s thoughts, feelings, and behavior are influenced roughly equally by the person’s body, mind, and psyche. It is possible to quantify both the processes within individuals and the external processes that affect their ways of adapting to life experiences. Changes in an individual’s thoughts, feelings, and behavior are dynamic expressions of complex adaptive systems that evolve gently from moment to moment and can be characterized not only by their means but also by their range. It is possible to identify specific clusters of genes and brain networks activated by the psychobiological processes underlying person-situation interactions and to show that these non-linear dynamic interactions are strongly correlated with individual differences in personality dimensions. Etcetera (C. R. Cloninger, 2006)	Cloninger’s Biopsychosocial Model	Temperament	**N**ovelty **S**eeking	The Tridimensional Personality Questionnaire (TPQ)	**NS:** I often try new things just for fun or thrills, even if most people think it is a waste of time.	Molecular Genetics
					**H**arm **A**voidance		**HA:** I often feel tense and worried in unfamiliar situations, even when others feel there is little to worry about.	Confirmatory Factor Analysis
					**R**eward **D**ependence	The Temperament and Character Inventory (TCI)	**RD:** I like to discuss my experiences and feelings openly with friends instead of keeping them to myself.	Non-Linear Factor Analysis
					**P**er**s**istence		**PS:** I often push myself to the point of exhaustion or try to do more than I really can.	Latent Profile Analysis
				Character	**S**elf-**D**irectedness	The Temperament and Character Inventory (TCI)	**SD:** In most situations, my natural responses are based on good habits that I have developed.	Latent Class Analysis
					**Co**operativeness		**CO:** I often consider another person’s feelings as much as my own.	
					**S**elf-**T**ranscendence	The Character Inventory	**ST:** I sometimes feel so connected to nature that everything seems to be part of one living organism.	

Note. + Character to some extent, especially prosocial traits, N/A Not Applicable.

## Data Availability

The datasets used and/or analyzed during the current study are available from the corresponding author on reasonable request.
